# Maternal smoking during pregnancy increases the risk of recurrent wheezing during the first years of life (BAMSE)

**DOI:** 10.1186/1465-9921-7-3

**Published:** 2006-01-05

**Authors:** Eva Lannerö, Magnus Wickman, Goran Pershagen, Lennart Nordvall

**Affiliations:** 1 Institute of Environmental Medicine, Karolinska Institutet, Stockholm, Sweden; 2 Department of Paediatrics, Karolinska University Hospital, Huddinge, Sweden; 3 Department of Occupational and Environmental Health, Stockholm County Council, Sweden; 4 Centre for Allergy Research, Karolinska Institutet, Stockholm, Sweden; 5 Department of Women's and Children's Health, Uppsala University, Uppsala, Sweden

## Abstract

**Background:**

Exposure to cigarette smoking during foetal and early postnatal life may have implications for lung health. The aim of this study was to assess the possible effects of such exposure in utero on lower respiratory disease in children up to two years of age.

**Methods:**

A birth cohort of 4089 newborn infants was followed for two years using parental questionnaires. When the infant was two months old the parents completed a questionnaire on various lifestyle factors, including maternal smoking during pregnancy and after birth. At one and two years of age information was obtained by questionnaire on symptoms of allergic and respiratory diseases as well as on environmental exposures, particularly exposure to environmental tobacco smoke (ETS). Adjustments were made for potential confounders.

**Results:**

When the mother had smoked during pregnancy but not after that, there was an increased risk of recurrent wheezing up to two years' age, OR_adj _= 2.2, (95% CI 1.3 – 3.6). The corresponding OR was 1.6, (95% CI 1.2 – 2.3) for reported exposure to ETS with or without maternal smoking in utero. Maternal smoking during pregnancy but no exposure to ETS also increased the risk of doctor's diagnosed asthma up to two years of age, OR_adj _= 2.1, (95% CI 1.2 – 3.7).

**Conclusion:**

Exposure to maternal cigarette smoking in utero is a risk factor for recurrent wheezing, as well as doctor's diagnosed asthma in children up to two yearsof age.

## Background

Many children are exposed to tobacco smoking, both before and after they are born. Maternal smoking during pregnancy is believed to affect the utero-placental flow, leading to an impaired foetal nutrition and consequent intrauterine growth retardation [1]. The foetus of smoking women is exposed from the time of conception to the same levels of nicotine as active smokers [2]. Smoking during pregnancy affects foetal lung development, reflected in spirometric flow in the neonate, especially when there is a family history of asthma and hypertension during pregnancy [3,4] and causes abnormal airway function [5,6]. Effects of ETS due to parental smoking on respiratory health in early childhood have been described in epidemiological studies [7-10] but few have made an effort to discriminate between effects of prenatal and postnatal exposure. Recent studies, however, suggest that smoke exposure in utero may be at least as detrimental to respiratory health in early life as postnatal exposure to ETS [11,12].

This prospective birth cohort study focuses on maternal smoking during pregnancy as a risk factor for recurrent wheezing during the first two years of life.

## Methods

### Study subjects

From February 1994 until November 1996, 4089 newborn infants (2,024 girls and 2,065 boys) were included in a population based prospective study, BAMSE (Children, Allergy, Milieu, Stockholm, Epidemiological survey). The children were born in predefined areas in Stockholm and recruited at their first visit to the Child Health Centre. During the recruitment period 7,221 infants were born in the study area and of these 1,256 were excluded because the families planned to move within a year, had insufficient knowledge of Swedish or an already enrolled older sibling. Another reason for exclusion was a serious disease in the neonate. For 477 infants correct addresses were not available. Thirteen hundred and ninety-nine declined participation. The final study cohort thus constituted 75 % of the eligible children. Details of the study design, inclusion criteria, enrolment and data collection are described in detail elsewhere [13-15].

### Questionnaire

The first questionnaire was filled in by the parents at the time of enrolment (Q0) at a median age of the children of 2 months (10^th ^percentile 0 months, 90^th ^percentile 5 months of age). The questionnaire aimed to assess the home environment as well as various indoor environmental exposures such as maternal smoking during pregnancy and smoking habits of both parents after birth of the child. A second part of the questionnaire covered the health of both parents with focus on allergic diseases i.e. asthma, allergic rhino-conjunctivitis and eczema. Socioeconomic status was classified according to the Nordic standard occupational classification (NYK) and Swedish socio-economic classification (SEI) [16]. The children were categorised on the basis of their parents' occupation into blue-collar workers, white-collar workers and others (students, unemployed). Identical questionnaires (Q1 and Q2) dealt with disease symptoms in the children and were distributed by mail to the parents when the children were one and two years of age. Combinations of reported symptoms were used to define criteria for different diagnoses (see below). Information on important exposure factors, such as parental smoking and breast-feeding, were also obtained from the questionnaires. The questions on symptoms and tobacco smoke exposure have been used in earlier studies [17-19]. Reminders for all three questionnaires were sent three times. The response rates to Q1 and Q2 were 96% and 94%, respectively. The median age for answering Q1 was 12 months and for Q2 24 months. Those who had responded to all three questionnaires (N = 3,791, 93%) before one, two and three years of age of the child, respectively, constituted the basis for this study.

### Assessment of pre- and postnatal tobacco smoke exposure

Foetal exposure to maternal smoking was reported in Q0 and was defined as maternal daily smoking of one cigarette or more during any trimester of pregnancy. The degree of such exposure was quantified for each trimester separately. Information on paternal smoking during the period in utero was not collected.

ETS was defined from exposure to maternal smoking of one cigarette or more daily during the first months of life and/or maternal smoking at one year of age of the child. Quantitative information i.e. the number of cigarettes smoked both of mothers and fathers, was obtained in Q0 for the first two months, Q1 and Q2 for the first and second year of life, respectively. In Q0 the parents also indicated whether they smoked at home and when the answer was yes whether they smoked on the balcony/at an open window/under the fan, thus actively avoiding direct exposure of the child.

### Classification of outcome

#### Recurrent wheezing up to two years of age

Three episodes of wheezing or more after three months of age in combination with the use of inhaled glucocorticoids and/or signs of bronchial hyperreactivity (wheezing or severe coughing when playing or being excited, or disturbed coughing at night not associated with common cold).

#### Doctor's diagnosed asthma

Reported "asthma" diagnosed by a doctor during the first and/or second year of life of the child.

#### Any wheezing

Wheezing and/or disturbing cough at night not associated with a common cold during the first and/or second year of life.

### Statistics

Odds ratios (ORs) and ninety-five percent confidence intervals (CIs) were calculated using logistic regression. To identify potential confounders several models including various covariates were tested (heredity, socioeconomy, maternal age, keeping of cat and/or dog, construction year of the home and duration of breastfeeding). Finally, adjustments were made for heredity (defined as doctor-diagnosed asthma and asthma medication and/or allergic rhino-conjunctivitis diagnosed by a doctor in combination with reported allergy to furred pets and/or pollen in one or both parents), exclusive breastfeeding during 4 months or more and maternal age ≥ 26 years, because these variables changed the OR estimates for smoking exposure. To test for interaction between smoking and other covariates an interaction term was included in the logistic regression model. The chi-square test and the Fisher exact test were used for statistical analyses of proportions.

Complete information on maternal smoking during pregnancy and answers on all three questionnaires were required to be included in the analyses and this was available for 3790 subjects.

Statistical analyses were made with the Stata Statistical Software: Release 8.0 (College Station, Texas, USA).

The study was approved by the ethical committee at the Karolinska Institutet, Stockholm, Sweden.

## Results

Short duration of breast-feeding, maternal age below 26 years, socio-economic status of the parents, the keeping of cat and/or dog and reported dampness were all associated with maternal smoking during pregnancy (table 1). In total, 469 infants were exposed to maternal smoking in utero. The prevalence of smoking decreased during pregnancy and reported smoking during the first, second and third trimester were 12%, 10 % and 9 % respectively. Twelve percent of the mothers reported to have smoked at least one cigarette daily during any part of or all through pregnancy. During the child's first two months the corresponding proportion was 8.0%, and when the child was one and two years old 9.4 and 10%, respectively. The corresponding reported postnatal exposure to paternal smoking was 16, 12 and 11%, respectively. Any exposure to ETS during the first two years of life of the children was reported for 25% of the children. In families with smoking fathers 34% of the mothers smoked compared to 8.3% in families with non-smoking fathers (p < 0.001). Most of the smoking parents (94%) reported in Q0 that they almost always smoked only outdoors, near open window or under the fan when at home.

**Table 1:** Characteristics of a cohort of children and their families by exposure to maternal daily smoking of one cigarette or more during pregnancy

	No foetal exposure			Foetal exposure to tobacco smoking			
	n/N^1^	%	95% CI	n/N^1^	%	95% CI	p-value^2^
Gender (male)	1676/3321	51	49 – 52	242/469	52	47 – 56	0.646
Parental asthma	533/3302	16	15 – 17	73/457	16	13 – 19	0.927
Birth weight <2500 g	121/3321	3.6	3.0 – 4.3	19/469	4.1	2.5 – 6.3	0.661
Gestational age <36 w	102/3321	3.1	2.5 – 3.7	15/469	3.2	1.8 – 5.2	0.946
Exclusive breastfeeding ≥4 months	2690/3316	81	80 – 82	323/469	69	65 – 73	<0.001
Maternal age ≥26 years	3091/3320	93	92 – 94	413/469	88	85 – 95	<0.001
Socioeconomic index (SEI)^3^							
1) Blue-collar	482/3311	15	13 – 16	140/468	30	26 – 34	<0.001
2) White collar	2792/3311	84	83 – 86	323/468	69	62 – 71	<0.001
3) Others^4^	37/3311	1.1	0.8 – 1.5	5/468	1.1	0.1 – 2.0	0.924
Keeping of cat and/or dog	426/3321	13	12 – 14	121/469	26	22 – 30	<0.001
Signs of dampness^5^	267/3309	8.1	7.1 – 9.0	39/468	8.3	5.8 – 11	0.844
Construction year of the home >1961	1679/3319	49	48 – 51	266/468	57	52 – 61	0.003

^1 ^The numbers do not add up because of missing data.

^2 ^Pearson chi-square test.

^3 ^Socioeconomic status of the parents according to socioeconomic index measured by the Nordic standard occupational classification (NYK) and Swedish socioeconomic classification (SEI).

^4 ^Student, unemployed etc.

^5^Smell or visible signs of mould in the dwelling and/or water damage inside construction.

**Figure 1:**
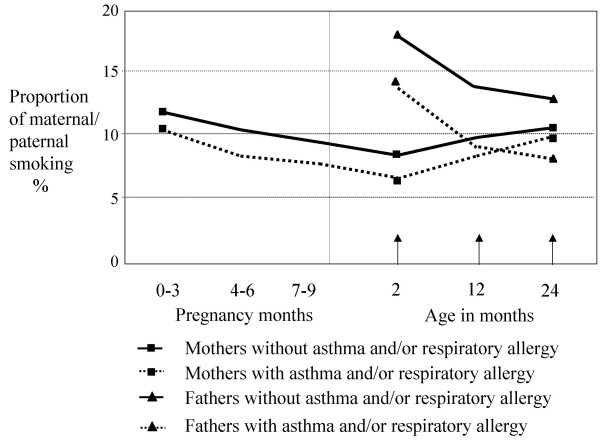
Smoking during pregnancy and the first two years of the child and parents with or without asthma and/or respiratory allergy.

The reported smoking of mothers with asthma or respiratory allergy (asthma requiring medication and/or doctor's diagnosed allergic rhino-conjunctivitis with reported allergy to furred pets and/or pollen) tended to be lower than that of mothers without such allergy both during pregnancy and the child's first two years (figure 1). This also held true for paternal smoking.

**Figure 2:**
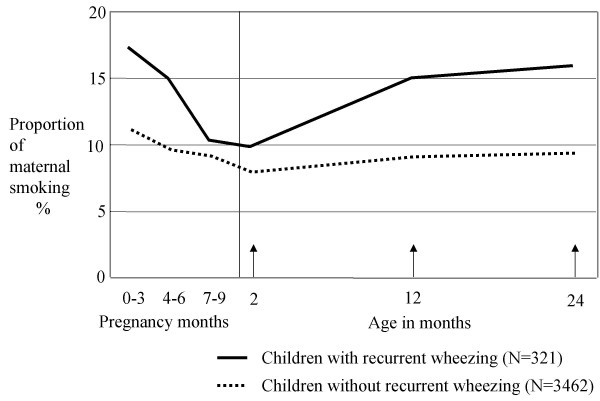
Proportion of maternal smoking of one or more cigarettes daily during pregnancy and during the first two years of the child among children with and without recurrent wheezing.

The cumulative incidence of recurrent wheezing, doctor's diagnosed asthma and any wheezing up to two years of age were 8.5%, 6.5% and 27%, respectively. The reported smoking pattern of mothers of children with recurrent wheezing differed from that of the mothers with children without recurrent wheezing (figure 2). Maternal smoking of one cigarette daily or more was reported for 16 % of the children with recurrent wheezing at one year of age, compared to 8.7% for healthy children (p < 0.001). The corresponding proportions at two year's age were 17 and 9.4% (p < 0.001). Eleven percent of the mothers of the children with recurrent wheezing reported to have smoked ten cigarettes or more daily at one and 12% at two years age. The corresponding figures were 6.3% and 7.0% for mothers with healthy children.

A large majority of infants (85%) were reported neither to have been exposed to maternal smoking during pregnancy, nor to any maternal smoking during the first two months of life and/or at one year of age, and these constituted the reference group. One-hundred and thirty-three children (3.6%) had been exposed in utero, but not after being born. Eleven percent of the children were exposed to ETS with or without maternal smoking in utero. Only 2.4% of the children were reported to have been exposed exclusively to ETS.

Maternal smoking during any period of pregnancy, but not after giving birth was associated with an increased risk of recurrent wheezing at two years of age, (OR_adj _= 2.2, 95% CI 1.3–3.6), (table 2). The effect appeared most pronounced when there was maternal smoking during the first and/or second trimester, (OR_adj _= 2.5, 95 % CI 1.5–4.0), but not thereafter in a separate analysis using the entire material and adjusting for the effect of ETS (data not shown).

**Table 2:** Recurrent wheezing, doctor's diagnosed asthma and any wheezing up to two years of age in relation to exposure to maternal smoking during pregnancy^6 ^and ETS^7 ^with or without maternal smoking during pregnancy.

	N	n	OR crude	95% CI	OR adj^8^	95 % CI
**Recurrent wheezing up to two years of age**						
No maternal smoking during pregnancy and no exposure to ETS^9^	3222	246	1		1	
Maternal smoking during pregnancy but no exposure to ETS	135	21	2.2	1.4 – 3.6	2.2	1.3 – 3.6
Exposure to ETS with or without smoking during pregnancy	422	54	1.8	1.3 – 2.4	1.6	1.2 – 2.3
						
**Doctor's diagnosed asthma up to two years of age**						
No maternal smoking during pregnancy and no exposure to ETS	3224	191	1		1	
Maternal smoking during pregnancy but no exposure to ETS	134	16	2.2	1.3 – 3.7	2.1	1.2 – 3.7
Exposure to ETS with or without smoking during pregnancy	424	37	1.5	1.1 – 2.2	1.4	0.95 – 2.1
						
**Any wheezing up to two years of age**						
No maternal smoking during pregnancy and no exposure to ETS	3207	855	1		1	
Maternal smoking during pregnancy but no exposure to ETS	135	50	1.6	1.1 – 2.3	1.7	1.2 – 2.4
Exposure to ETS with or without smoking during pregnancy	422	142	1.4	1.1 – 1.7	1.3	1.0 – 1.6

^6 ^Maternal smoking of one cigarette a day or more.

^7 ^Maternal tobacco smoking during the first months of life and/or at one year of life.

^8 ^Adjusted for heredity, defined as asthma and/or allergic rhino- conjunctivitis diagnosed by a doctor and in combination with reported allergy to furred pets and/or pollen in one or both parents (reported asthma medication was required for asthma diagnosis), maternal age and length of exclusive breast feeding.

^9 ^Reference category: no maternal smoking during pregnancy and no exposure to ETS.

Exposure to ETS alone or in combination with exposure in utero tended to be associated with an increased risk of recurrent wheezing (OR_adj _= 1.6, 95 % CI 1.2 – 2.3). The risk estimates were similar in the different exposure groups for doctor's diagnosed asthma and any wheezing up to two years of age, respectively (table 2). These effects were independent of gender of the infant (data not shown).

Exposure to cigarette smoking during pregnancy and of maternal smoking during the child's first year of life increased the risk of recurrent wheezing as well as of doctor's diagnosed asthma and any wheezing, respectively, at one year of age, in a similar way as reported in table 2. Reported paternal smoking during the child's first year of life had no additional effect on any of the outcomes under study (data not shown).

The results of dose-response analyses were not conclusive i.e. neither confirmed nor excluded a trend, mainly due to low numbers of subjects in the high exposure groups (data not shown). Furthermore, there was no clear evidence of interaction between smoking and heredity or gender (data not shown).

## Discussion

This study provides strong evidence that exposure in utero to maternal smoking is important for development of recurrent wheezing during the first two years of life, irrespective of exposure to ETS after birth. Similar results have been published by others, but generally without separating the effects of exposure in utero exposure to ETS during the first few years of life [20,21]. The study by Lux and coworkers, however, clearly indicates that maternal smoking restricted to pregnancy causes wheezing [11]. The design of their study is similar to ours and allows for separation of the effects of different exposure periods but data about smoking during pregnancy were only obtained for gestational weeks 30–32. In the present study information about maternal smoking during pregnancy encompassed the various trimesters in detail. Our data suggest an effect with exposure particularly during early pregnancy. If so, this is possibly a consequence of an effect on intra-uterine growth [1].

An effect of maternal smoking on the foetus has also been documented by several studies of pulmonary function in neonates [4,6,22,23]. Most of these studies indicate hampered expiratory flows as indices of a detrimental effect. In a study by Hoo and co-workers prematurely born infants, in average seven weeks, were investigated and maternal smoking was associated with reduced pulmonary function [24]. The spirometric data in neonates only give indirect evidence of a reduction in airway diameter. For obvious reasons no direct studies of morphological consequences of exposure to smoking in the neonate lung have been carried out in healthy term babies. However, in children with sudden infant death increased airway thickness has been associated with maternal smoking of more than 20 cigarettes daily [25]. To which extent this effect stems from exposure prior to or after birth is not clear.

In many studies the role of ETS, as a determinant of childhood asthma, has been investigated but in most of them without due consideration of the separate influence of maternal smoking during pregnancy [8,26]. In a meta-analysis by Strachan and Cook a pooled risk estimate of 1.57 was found for lower respiratory illness in relation to smoking by either parent [7]. The relative contributions of pre- and postnatal smoking were not disentangled. In the study by Lux, an OR of 1.3 was found for exposure to ETS exclusively [11]. Possibly, the effect of exposure in utero may be the more important which is also supported by our data.

In Sweden exposure of children to tobacco smoking has been reduced to levels which are low in an international perspective. This is probably a consequence of a very active health policy and an effective maternal and child health care. During the study there was also a campaign "Smokefree children" through the Child Health Centres which reached almost all (99.5%) of the families when the baby was new-born (Statistics from Child Health Centres, Stockholm County Council, 1995). The effects of ETS are possibly diminished because of an overall awareness of the detrimental effects of exposure. This is supported by the finding that 94% of the parents reportedly never exposed their children to ETS. Exposure of the foetus, on the other hand, cannot be avoided by the pregnant mothers who are active smokers.

Participation in the study is most likely to have been affected by parental awareness of health hazards associated with cigarette smoking. Thus, smokers may to a higher extent than non-smokers have chosen not to join the study. A study of non- responders and actively excluded families of the BAMSE study showed that these parents smoked more than those included in the cohort [15]. This would render the study base less representative of the population, but in relation to tobacco smoke exposure probably not affect the risk estimate of smoking related health effects. Furthermore, parents with allergic diseases would possibly be more willing to join the original cohort but we found no such selection. We had the advantage of a large sample, allowing for the assessment of effects of exposures in subgroups of infants. Yet, possible biases must be taken into account. Smoking tobacco was found to be associated with a negative family history of allergic disease. Furthermore, we based the risk estimation on maternal smoking only, for obvious reasons regarding smoking in pregnancy, but this may lead to some misclassification of exposure postnatally. The effects of the role of ETS will be studied more in detail in the future follow up if the cohort.

The main implication of this study is that smoking cessation programmes need to be targeted on childbearing ages. In maternal health care such efforts should focus not only on those who are already pregnant, but also on women who plan to conceive.

## Competing interests

The author(s) declare that they have no competing interests.

## Authors' contributions

All four authors have made substantial intellectual contributions to this study and have also been involved in the BAMSE project since it started.
